# Arterial calcification at multiple sites: sex-specific cardiovascular risk profiles and mortality risk—the Rotterdam Study

**DOI:** 10.1186/s12916-020-01722-7

**Published:** 2020-09-24

**Authors:** Janine E. van der Toorn, Oscar L. Rueda-Ochoa, Niels van der Schaft, Meike W. Vernooij, M. Arfan Ikram, Daniel Bos, Maryam Kavousi

**Affiliations:** 1 Department of Epidemiology, University Medical Centre, Erasmus MC, PO Box 2040, 3000 CA Rotterdam, The Netherlands; 2 Department of Radiology and Nuclear Medicine, University Medical Centre, Rotterdam, The Netherlands; 3 Electrocardiography Research Group, School of Medicine, Industrial University of Santander, Bucaramanga, Colombia; 4 Department of Clinical Epidemiology, Harvard T.H. Chan School of Public Health, Boston, USA

**Keywords:** Arteriosclerosis, Calcification, Computed tomography, Mortality, Risk profiles

## Abstract

**Background:**

Evidence has pointed towards differences in the burden of arteriosclerosis according to its location and sex. Yet there is a scarcity of population-based data on aggregated sex-specific cardiovascular risk profiles, instead of single risk factors, and mortality risk according to the location of arteriosclerosis. We assessed sex-specific cardiovascular risk profiles and mortality risk associated with arteriosclerosis.

**Methods:**

From the population-based Rotterdam Study, 2357 participants (mean age 69 years, 53% women) underwent non-contrast computed tomography to quantify calcification, as a proxy for arteriosclerosis, in the coronary arteries (CAC), aortic arch (AAC), extracranial (ECAC) and intracranial carotid arteries (ICAC), vertebrobasilar arteries (VBAC), and aortic valve (AVC). Principal component analysis (PCA) of eight distinct cardiovascular risk factors was performed, separately for women and men, to derive risk profiles based on the shared variance between factors. We used sex-stratified multivariable logistic regression to examine the associations between PCA-derived risk profiles and severe calcification at different locations. We investigated the associations of severe calcification with mortality risk using sex-stratified multivariable Cox regression.

**Results:**

PCA identified three cardiovascular risk profiles in both sexes: (1) anthropometry, glucose, and HDL cholesterol; (2) blood pressure; and (3) smoking and total cholesterol. In women, the strongest associations were found for profile 2 with severe ECAC and ICAC (adjusted OR [95% CI] 1.32 [1.14–1.53]) and for profile 3 with severe at all locations, except AVC. In men, the strongest associations were found for profile 2 with VBAC (1.31 [1.12–1.52]) and profile 3 with severe AAC (1.28 [1.09–1.51]). ECAC and AVC in women and CAC in men showed the strongest, independent associations with cardiovascular mortality (HR [95% CI] 2.11 [1.22–3.66], 2.05 [1.21–3.49], 2.24 [1.21–3.78], respectively).

**Conclusions:**

Our findings further underline the existence of sex- and location-specific differences in the etiology and consequences of arteriosclerosis. Future research should unravel which distinct pathological processes underlie differences in risk profiles for arteriosclerosis.

## Background

Arteriosclerosis is considered the most important condition underlying cardiovascular events [1]. Although arteriosclerosis occurs systemically throughout the arterial system, increasing evidence points towards distinct artery-specific differences across individuals which is particularly striking when comparing women and men [2–5].

These differences in the burden of arteriosclerosis across arteries and between women and men could be of pathophysiological origin but are also likely to reflect underlying differences in modifiable cardiovascular risk factors. To date, studies focused largely on single cardiovascular risk factors [3, 6–9]. However, cardiovascular risk factors tend to aggregate, interact, and persist in combination [10]. Studying risk profiles in an aggregated manner would allow to account for the interaction between various cardiovascular risk factors [11, 12]. This could provide insight into the etiology of arteriosclerosis and may eventually contribute to facilitate risk stratification in clinical settings.

Similarly, arteriosclerosis may vary across locations and sex with regard to its contribution to mortality risk. While location-specific discrepancies in mortality risk associated with arteriosclerosis have been reported [2, 13, 14], sex-specific mortality remains poorly addressed. Within a large prospective population-based cohort, we investigated sex-specific cardiovascular risk profiles associated with arterial calcification, as a proxy for arteriosclerosis, at six different locations. We further examined sex- and location-specific associations of calcification with the risk of mortality.

## Methods

### Study population

This study is embedded within the Rotterdam Study, a large prospective population-based cohort among adults and elderly living in the Ommoord district in Rotterdam, comprising over 15,000 participants. Each participant was examined at study entry, and follow-up examinations were carried out every 3 to 5 years. Between 2003 and 2006, a random sample of 3229 participants visiting the research center were invited to undergo a multidetector computed tomography (MDCT) scan to visualize arterial calcification, as a proxy for arteriosclerosis, at the following six locations: coronary arteries, aortic arch, extracranial carotid arteries, intracranial carotid arteries, vertebrobasilar arteries, and aortic valve. In total, 2524 participants were scanned (response rate, 78%). Out of 2524 scans, 167 were not gradable for arterial calcification at any of the six locations because of the presence of a coronary stent, pacemaker, or image artifacts, leaving a total of 2357 complete examinations with information on calcification at all six locations.

The Rotterdam Study has been approved by the Medical Ethics Committee of the Erasmus MC (registration number MEC 02.1015) and by the Dutch Ministry of Health, Welfare and Sport (Population Screening Act WBO, license number 1071272-159521-PG). The Rotterdam Study has been entered into the Netherlands National Trial Register (NTR; www.trialregister.nl) and into the WHO International Clinical Trials Registry Platform (ICTRP; www.who.int/ictrp/network/primary/en/) under shared catalogue number NTR6831. All participants provided written informed consent to participate in the study and to have their information obtained from treating physicians.

### Assessment of cardiovascular risk factors

Data collection of cardiovascular risk factors included a standardized home interview and visits to the research centre for physical examination and blood sampling [15]. Body mass index (BMI) was calculated as weight (kg)/height (m)^2^. Waist circumference was measured between the lower rib margin and the iliac crest while in a standing position. Hip circumference was obtained as the maximum circumference over the buttocks. Accordingly, the waist-to-hip ratio (WHR) was calculated as the ratio of waist circumference over the hip circumference. Systolic and diastolic blood pressures were measured twice at the right arm using a random-zero sphygmomanometer, and the average of the measurements was used. Pulse pressure was calculated subtracting diastolic blood pressure from systolic blood pressure. Serum total cholesterol and high-density lipoprotein (HDL) cholesterol were assessed using an automatic enzymatic procedure (Hitachi 911, Roche CHOD PAP). Glucose levels were determined enzymatically by the hexokinase method. Information on antidiabetic therapy, blood pressure- and lipid-lowering medication use, and smoking behavior including amounts of cigarettes per day was obtained by trained interviewers. Subsequently, smoking behavior was categorized as “current smoking” and “non-smoking”. We defined history of cardiovascular disease as a history of myocardial infarction (MI), stroke, percutaneous transluminal coronary angioplasty (PCI), and/or coronary artery bypass graft (CABG). Information on MI, stroke, PCI, and CABG was obtained at baseline and during follow-up visits as described previously [16–18].

### Assessment of calcification

We used 16-slice or 64-slice MDCT scanners (Somatom Sensation 16 or 64; Siemens, Forchheim, Germany) to obtain non-contrast CT images. We performed a cardiac scan and a scan that ranged from the aortic root to the Circle of Willis. On these scans, we assessed calcification in the coronary arteries, aortic arch, extracranial and intracranial carotid arteries, vertebrobasilar arteries, and the aortic valve. To control the timing of the image acquisition and synchronize with cardiac motion, cardiac images were obtained within a single breath-hold and reconstructed with 12 mm × 1.5 mm collimation, 120 kVp, effective 30 mAs, and prospective ECG triggering at 50% of the cardiac cycle. If the heart rate was irregular during breath-holding, the images were obtained with 12 mm × 0.75 mm collimation, 150 effective mAs, and retrospective ECG gating [3]. Coronary artery calcification (CAC), aortic arch calcification (AAC), extracranial carotid artery calcification (ECAC), and aortic valve calcification (AVC) were quantified using dedicated software (Syngo Calcium Scoring; Siemens, Forcheim, Germany). Intracranial carotid artery calcification (ICAC) and vertebrobasilar artery calcification (VBAC) were quantified using a semiautomatic scoring method that allows to manually segment calcification in each consecutive CT slice after which the calcification volume is computed using the pixel size and the increment [2, 19, 20]. A detailed description of the evaluation methods is provided elsewhere [2, 3, 19–21].

### Assessment of mortality

Information on vital status was obtained through municipal records and continuous digital linkage with general practitioner files [18]. Data on cause of death were obtained from general practitioners and hospital records. Research physicians adjudicated all deaths according to the International Classification of Diseases, 10th revision (ICD-10) codings as described in detail previously [18]. For the current study, causes of deaths were categorized as cardiovascular deaths and non-cardiovascular deaths according to the ICD-10 codings. The ICD-10 codes associated with cardiovascular diseases were I21, I25, I26, I33, I35, I42, I46, I50, I61-I64, I69, I69, I70, I71, I73, I74, and I99. Follow-up for mortality was complete until January 1, 2015.

### Statistical analysis

Descriptive characteristics of the study population are shown in means ± standard deviations or medians with interquartile ranges (IQR) for continuous variables and in absolute numbers and percentages for categorical variables. Differences in study population characteristics between women and men were estimated using the Mann-Whitney *U* test (non-parametric variables), *t* test (parametric variables), and chi-square test. Using Spearman’s correlation coefficient, we calculated the sex-specific correlations of calcification volumes across the six locations. We performed a non-parametric bootstrap procedure with 1000 replications to obtain interval estimates of Spearman’s rho.

We examined the associations between cardiovascular risk profiles and calcification at different locations using the following strategy. First, to empirically derive risk factor profiles with a common and unique high shared variance, cardiovascular risk factors (BMI, WHR, smoking (cigarettes per day), systolic blood pressure, pulse pressure, glucose, total and HDL cholesterol) were submitted to a principal component analysis (PCA) using the varimax rotation method to produce interpretable components, i.e., cardiovascular risk profiles, for women and men separately [22]. Components with eigenvalues above 1 were considered, and subsequently, a factor score for each individual was computed weighted by the factor loadings. These factor scores were then standardized using a *z*-score scale in order to quantify its contribution to the risk factor component. Sex-specific quartiles of calcification volumes per location were computed, and the highest quartile was defined as severe calcification. For VBAC, the prevalence was only 20%, precluding the possibility of quartile analyses. Hence, for VBAC, we used the presence of calcification in the analyses. Second, using logistic regression modeling, we examined associations of the PCA-derived cardiovascular risk profiles with severe CAC, AAC, ECAC, ICAC, VBAC, and AVC, while adjusting for age, cohort, scanner, medication use (antidiabetic therapy, blood pressure- and lipid-lowering medication), and history of cardiovascular disease.

Next, we investigated the association of severe calcification at each location and the risk of all-cause, cardiovascular, and non-cardiovascular mortality using Cox regression. To this end, a model was fitted along with age, cohort, scanner, BMI, systolic blood pressure, diastolic blood pressure, smoking status, glucose, total and HDL cholesterol, medication use (antidiabetic therapy, blood pressure- and/or lipid-lowering medication), and history of cardiovascular disease. To investigate whether associations of severe calcification at each location with mortality risk are independent of severe calcification at other locations, we also entered all locations simultaneously into one model, adjusted for age, cohort, and scanner. The proportional hazard assumption was met for all analyses (all *P* values > 0.05). In all analyses, we checked whether age squared improved the models, but as the coefficients did not change after adding age squared, we adjusted only for age. Analyses were repeated in participants without a history of cardiovascular disease. All analyses were stratified by sex.

To account for missing information on covariables (maximum amount of missingness was 2.6%), we used multiple imputation by chained equation [23]. Analyses were performed using SPSS Statistics 24 (IBM, Chicago, IL, www.spss.com), R (R Foundation for Statistical Computing, Vienna, Austria, URL http://www.R-project.org/), and RStudio 3.4.4 (Boston, MA, USA, URL http://www.rstudio.org/).

## Results

Baseline characteristics of the study population are shown in Table 1. The mean age of the 2357 participants was 69 years, and 53% were women. The prevalence and median volume of calcification at all locations were higher in men than in women. Calcification was most prevalent in the aortic arch and least prevalent in the vertebrobasilar arteries in both sexes.

**Table 1:** Descriptive characteristics of the study population according to sex

	Women, *N* = 1239	Men, *N* = 1118	*P* value*
Age (years)	69.5 (6.9)	69.6 (6.6)	0.606
BMI (kg/m^2^)	27.8 (4.4)	27.4 (3.5)	0.010
Waist-to-hip ratio	0.9 (0.1)	1.0 (0.1)	< 0.001
Systolic blood pressure (mmHg)	147.3 (20.6)	146.1 (19.6)	0.151
Diastolic blood pressure (mmHg)	79.2 (10.6)	81.4 (10.8)	< 0.001
Pulse pressure (mmHg)	68.1 (17.8)	64.7 (16.8)	< 0.001
Total cholesterol (mmol/L)	5.9 (1.0)	5.4 (0.9)	< 0.001
HDL cholesterol (mmol/L)	1.6 (0.4)	1.3 (0.3)	< 0.001
Glucose (mmol/L)	5.7 (1.3)	5.8 (1.2)	0.006
Current smoking	169 (13.6)	196 (17.4)	0.009
Use of lipid-lowering medication	295 (23.8)	254 (22.6)	0.383
Use of blood pressure-lowering medication	482 (38.9)	458 (41.0)	0.346
Antidiabetic therapy	77 (6.2)	80 (7.2)	0.406
History of cardiovascular disease	74 (6.0)	157 (14.0)	< 0.001
Presence of calcification			
CAC	923 (74.5)	1010 (90.3)	< 0.001
AAC	1140 (92.0)	1044 (93.4)	0.202
ECAC	836 (67.5)	889 (79.5)	< 0.001
ICAC	1001 (80.8)	925 (82.7)	0.222
VBAC	223 (18.0)	254 (22.7)	0.004
AVC	332 (26.8)	436 (39.0)	0.001
Volumes of calcification			
CAC (mm^3^)^a^	16.5 (0.0–117.9)	132.2 (18.3–485.5)	< 0.001
AAC (mm^3^)^a^	226.9 (40.7–805.6)	281.9 (51.7–949.2)	0.073
ECAC (mm^3^)^a^	12.4 (0.0–76.4)	41.3 (1.9–153.5)	< 0.001
ICAC (mm^3^)^a^	35.4 (5.5–118.0)	50.6 (9.2–172.3)	0.001
VBAC (mm^3^)^a^	0.0 (0.0–0.0)	0.0 (0.0–0.0)	0.005
AVC (mm^3^)^a^	0.0 (0.0–6.3)	0.0 (0.0–42.7)	< 0.001

Values are in means (standard deviations) for continuous variables, and absolute numbers (percentages) for categorical variables

*CAC* coronary artery calcification, *AAC* aortic arch calcification, *ECAC* extracranial carotid artery calcification, *ICAC* intracranial carotid artery calcification, *VBAC* vertebrobasilar artery calcification, *AVC* aortic valve calcification

^a^Median (interquartile range)

*P value of characteristic differences between men and women estimated using the *t* test or Mann-Whitney *U* test for (skewed) continuous variables and the chi-square test for categorical variables

### Correlation of calcification across different locations

**Fig. 1:**
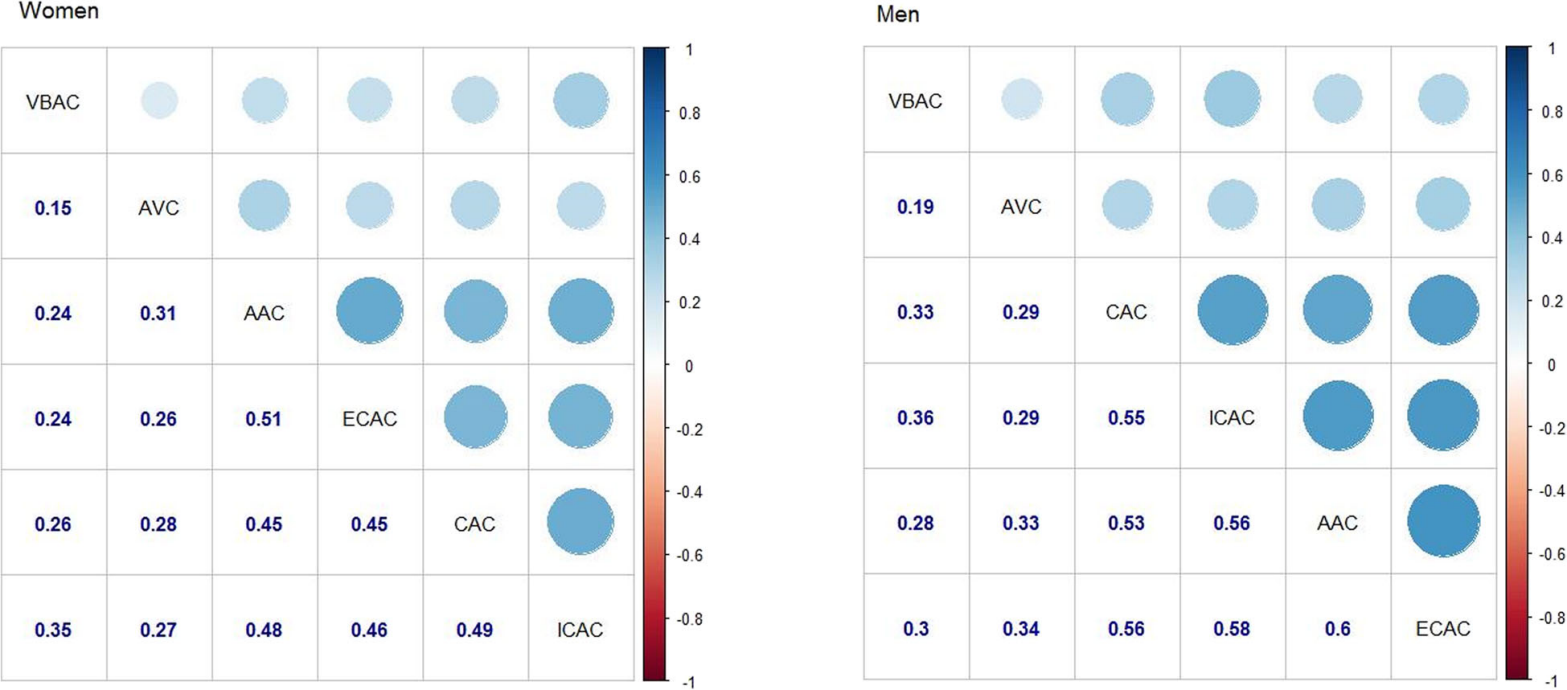
Spearman’s correlation coefficients for calcification at different locations among women and men. *P* < 0.001 for all correlations. Correlation coefficients are based on calcification volumes (mm^3^)

We found weak to moderate correlations of calcification volumes across the six locations. Correlations were slightly weaker among women (Fig. 1). The strongest correlation was found between AAC and ECAC (women: *r* = 0.51 [95% CI, 0.46–0.55]; men: *r* = 0.60 [95% CI, 0.55–0.64]) and the weakest between VBAC and AVC (women: *r* = 0.15 [95% CI, 0.09–0.21]; men: *r* = 0.19 [95% CI, 0.13–0.25]). Overall, VBAC and AVC showed the weakest correlations with other locations.

### Risk factor profiles of calcification

In both sexes, we identified three principal cardiovascular risk profiles, explaining over 60% of the total variance in the data (Additional file 1: TableS1). In the first risk profile, the dominant factors included were BMI, WHR, glucose, and HDL cholesterol. The second risk profile comprised systolic blood pressure and pulse pressure. In the third risk profile, the most dominant factors were smoking and total cholesterol.

**Fig. 2:**
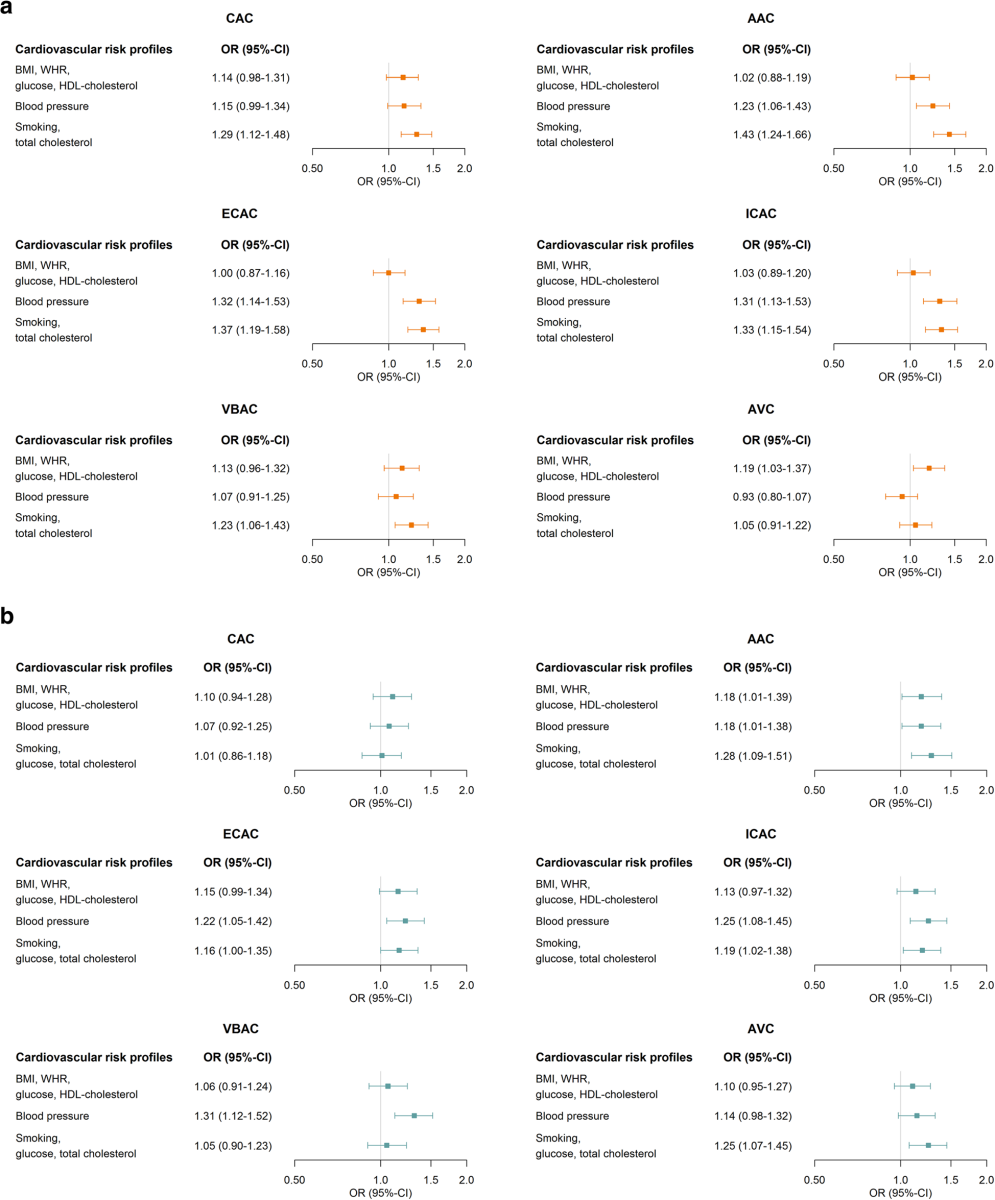
**a** Cardiovascular risk profiles and calcification at different locations, among women. **b** Cardiovascular risk profiles and calcification at different locations, among men. Values represent odds ratio and 95% confidence intervals (OR (95% CI)) for severe versus non-severe CAC, AAC, ECAC, ICAC, and AVC and for the presence of VBAC. Associations are adjusted for age, cohort, scanner, antidiabetic therapy, blood pressure, and/or lipid-lowering medication use, and history of cardiovascular disease

Figure 2 shows the multivariable-adjusted associations between the cardiovascular risk profiles and severe calcification volumes at all six locations. In women, the first risk profile was associated with severe AVC (OR 1.19 [95% CI, 1.03–1.37]), but not with calcification at other locations. The second risk profile in women was most prominently associated with severe ECAC and ICAC (OR [95% CI] 1.32 [1.14–1.53] and 1.31 [1.13–1.53], respectively), and the third risk profile was strongly associated with calcification at all locations, except AVC. In men, the first risk profile was associated with severe AAC only (OR 1.18 [95% CI, 1.01–1.39]). The second risk profile in men was most strongly associated with severe ECAC and ICAC and with the presence of VBAC (OR [95% CI] 1.22 [1.05–1.42], 1.25 [1.08–1.45], 1.31 [1.12–1.52], respectively). For the third risk profile, the largest magnitude was found for the association with severe AAC (OR 1.28 [95% CI, 1.09–1.51]).

### Risk of mortality

During 21,436 person-years of follow-up, 193 women and 259 men died (overall mortality rate 21.1 per 1000 person-years). Of these, 64 women and 70 men died due to cardiovascular causes. Table 2 shows the associations of severe calcification at the different locations with the risk of all-cause mortality, cardiovascular mortality, and non-cardiovascular mortality. In women, severe AAC and ECAC were associated with all-cause mortality (hazard ratio (HR) for the upper versus lowest three quartiles: 1.43 [95% CI, 1.04–1.97], 1.46 [95% CI, 1.07–2.00], respectively). In men, severe AAC and ECAC and the presence of VBAC were the strongest risk factors for all-cause mortality (HR [95% CI] 1.60 [1.21–2.10], 1.43 [1.10–1.86], 1.43 [1.10–1.88], respectively). Considering cardiovascular mortality in women, the most prominent associations were found for severe ECAC and AVC with the risk of cardiovascular mortality (HR 2.11 [1.22–3.66] and 2.05 [1.21–3.49], respectively). In men, the largest effect estimates were found for severe CAC and ICAC with the risk of cardiovascular mortality (HR [95% CI] 2.24 [1.33–3.78] and 2.11 [1.27–3.52], respectively). In addition, in men, severe AAC and the presence of VBAC were associated with the risk of non-cardiovascular mortality (HR [95% CI] 1.48 [1.07–2.04] and 1.48 [1.08–2.03], respectively). In the analysis including all locations in one model, the strongest, statistically significant associations were found for severe ECAC and AVC in women and CAC in men with cardiovascular mortality risk (Additional file 1: TableS2). Analyses among participants without a history of cardiovascular disease revealed similar results (Additional file 1: TablesS3-4, Additional file 1: Fig. S1).

**Table 2:** Calcification at different locations and the risk of mortality among women and men

	All-cause mortality	Cardiovascular mortality	Non-cardiovascular mortality
Women	(*N*_cases_/*N*_atrisk_ = 193/1239)	(*N*_cases_/*N*_atrisk_ = 64/1239)	(*N*_cases_/*N*_atrisk_ = 129/1239)
CAC	1.33 (0.96–1.82)	1.35 (0.78–2.35)	1.31 (0.89–1.94)
AAC	1.43 (1.04–1.97)	1.39 (0.80–2.43)	1.45 (0.98–2.15)
ECAC	1.46 (1.07–2.00)	2.11 (1.22–3.66)	1.22 (0.82–1.80)
ICAC	1.33 (0.95–1.86)	1.95 (1.08–3.51)	1.11 (0.73–1.67)
VBAC	1.23 (0.89–1.70)	1.28 (0.74–2.22)	1.21 (0.81–1.80)
AVC	1.15 (0.85–1.57)	2.05 (1.21–3.49)	0.85 (0.57–1.26)
Men	(*N*_cases_/*N*_atrisk_ = 259/1118)	(*N*_cases_/*N*_atrisk_ = 70/1118)	(*N*_cases_/*N*_atrisk_ = 189/1118)
CAC	1.36 (1.04–1.79)	2.24 (1.33–3.78)	1.13 (0.82–1.58)
AAC	1.60 (1.21–2.10)	2.02 (1.19–3.43)	1.48 (1.07–2.04)
ECAC	1.43 (1.10–1.86)	1.74 (1.06–2.85)	1.34 (0.98–1.84)
ICAC	1.34 (1.03–1.75)	2.11 (1.27–3.52)	1.13 (0.83–1.56)
VBAC	1.43 (1.10–1.88)	1.34 (0.80–2.23)	1.48 (1.08–2.03)
AVC	1.20 (0.92–1.56)	1.52 (0.94–2.48)	1.10 (0.78–1.50)

Adjusted for age, cohort, scanner, body mass index, systolic blood pressure, diastolic blood pressure, smoking status, glucose, total cholesterol, HDL cholesterol, antidiabetic therapy, blood pressure, and/or lipid-lowering medication use, and history of cardiovascular disease. Values represent hazard ratios (95% confidence intervals) for a higher burden of each component and for the upper quartile versus the lowest three quartiles (CAC, AAC, ECAC, ICAC, AVC) or the presence of calcification (VBAC)

*CAC* coronary artery calcification, *AAC* aortic arch calcification, *ECAC* extracranial carotid artery calcification, *ICAC* intracranial carotid artery calcification, *VBAC* vertebrobasilar artery calcification, *AVC* aortic valve calcification

## Discussion

Our study revealed three cardiovascular risk profiles that showed heterogeneous associations with arteriosclerosis according to location and sex. Among women, the strongest associations were found for the risk profile characterized by smoking and total cholesterol with severe calcification at all sites, except the aortic valve. Among men, the strongest association was found for the risk profile characterized by blood pressure measures with the presence of VBAC. Calcification in the coronary arteries among men and in the aortic valve and extracranial carotid arteries among women showed the strongest associations with cardiovascular mortality, independent of calcification at other locations.

Our study provides insight into sex-specific clustering of risk factors and the relation with arteriosclerosis. The extent to which single risk factors contribute to the risk profiles—as reflected in the factor loadings of each risk factor—demonstrates important differences between women and men. While current interventions for cardiovascular risk reduction focus mainly on single risk factors, our study demonstrates the important interaction between single risk factors translating into differences in the risk of arteriosclerosis.

In women, we found no association of the first risk profile, characterized by anthropometry, glucose, and HDL cholesterol, with calcification at any location except for the aortic valve. Arguably, the anthropometric element of the risk profile may weaken the association of the profile with calcification. For example, estrogen production in fat mass in women may lead to favorable alteration of the lipoprotein profiles and a reduced expression of adhesion molecules [24–26]. Considering the second risk profile in women, i.e., the blood pressure profile, we found the strongest associations with calcification in both extracranial and intracranial carotid arteries. In accordance with our findings, hypertension has been identified as a strong risk factor for calcification in the carotid arteries in women previously [3]. Interestingly, the third risk profile in women, representing smoking and total cholesterol, was associated with calcification in all vessels, but not with calcification in the aortic valve. Although we encourage further research into this topic, we hypothesize the apparent distinct role of the aortic valve may be found in different pathophysiological mechanisms of calcific aortic valve leaflets compared to that of the vasculature.

In men, the first risk profile, representing anthropometric measures, glucose, and HDL cholesterol, was associated with severe AAC only. A large autopsy study among individuals aged 15–34 years of which 75% were men demonstrated synergistic interaction between glucose and cholesterol with consequent adverse effects on arteriosclerosis in both the coronary and aortic arteries [27]. Despite inconsistencies in the literature, especially with regard to anthropometric measures [26, 28–30], we may argue that the redistribution of fat with advancing age towards more visceral fat, which is poorly captured in anthropometric measures, might explain the less pronounced associations between the first profile and calcification [31].

With regard to the second profile in men, hypertension has been established as an important risk factor for arteriosclerosis at different locations [20, 29]. In our study, the second profile in men was most strongly associated with severe ICAC and ECAC, and with the presence of VBAC, while we found less pronounced associations with other locations. A possible mechanism underlying the differential vulnerability to blood pressure could pertain to anatomical differences in terms of curvature (branching) or diameter resulting in the varying extent of hemodynamic forces and arterial wall shear stress across different segments of the vasculature [11].

The third profile in men, characterized by smoking, glucose, and total cholesterol, was most prominently associated with severe AVC and AAC. These factors have been associated with aortic arteriosclerosis in men previously [32, 33].

In addition to the heterogeneity in cardiovascular risk profiles associated with calcification, we also found differing associations of calcification in various vessels with mortality risk. The effect of arteriosclerosis as a systemic process is reflected in the effect estimates for mortality that slightly attenuated after adjustments for calcification in all vessel beds. However, the most prominent findings regarding the association of ECAC and AVC in women and CAC in men with cardiovascular mortality remained strong and statistically significant, irrespective of calcification in other vessel beds. This underpins our arguments on the existence of important locally driven effects. In line with our study, carotid arteriosclerosis has been established a strong predictor for cardiovascular mortality in women [34]. Previous studies also reported strong associations of AVC with mortality and cardiovascular events [35, 36]. However, to our knowledge, no previous population-based studies have focused on sex-specific associations of calcification in the aortic valve with cardiovascular mortality.

Another noteworthy finding was the association of VBAC with mortality among men. Despite its potential detrimental effects on the brain, VBAC has not been studied in relation to mortality in a population-based setting. Yet, in a sample of patients with symptomatic vertebrobasilar stenosis, a low stroke-free survival rate has been reported [37]. We found that VBAC was a strong independent indicator for mortality in men, which was especially driven by non-cardiovascular causes of death. Arguably, neurodegenerative diseases could play an important role in the association between VBAC and non-cardiovascular causes of death. Calcification in other vessels, e.g., within the intracranial and extracranial carotid arteries, has been associated with a higher risk of dementia, cognitive decline, and unfavorable brain changes [38, 39]. Finally, in line with previous literature, we found that AAC and ECAC were strongly associated with the risk of all-cause mortality irrespective of sex [2, 40].

Our study showed sex-specific aggregation of cardiovascular risk factors resulting in differential contributions to arteriosclerosis at various sites. Future studies are needed to provide a more in-depth understanding of the interaction between co-occurring risk factors and how they convey changes of arteriosclerosis over time. The combined effect of cardiovascular risk factors may carry clinically relevant information which might eventually lead to improvements in current practice such as the development of risk profile-dependent preventive strategies for cardiovascular risk reduction. Also, it is important to understand which pathophysiological processes underlie the differences in cardiovascular risk profiles and consequences of arteriosclerosis. Therefore, it would be valuable to link risk profile-specific adverse effects as measured by, for example, shear stress, inflammation, and endothelial function with longitudinal measures of arteriosclerosis.

### Strengths and limitations

Strengths of our study include the population-based setting and the large sample size with calcification assessment in various vessels within the same individuals which allows head-to-head comparisons. Yet several methodological considerations should also be noted. First, the use of CT allows to visualize calcification only. Hence, any non-calcified components of the disease were not visualized. Nonetheless, the amount of calcium adequately reflects the underlying arteriosclerotic disease [41]. Second, despite our large population with calcification assessment in various vessels, borderline significant findings, for example, with regard to risk profiles for CAC in women and AVC in men may have resulted in under-recognition of potentially important associations. Third, as the derivation of the risk profiles has a data-driven nature, its validity might be dependent on the study population.

## Conclusions

Our study revealed three cardiovascular risk profiles in both sexes that were differentially associated with calcification at multiple vascular sites. Moreover, heterogeneous associations between calcification at different locations and mortality were observed. ECAC and AVC in women and CAC in men showed the strongest associations with cardiovascular mortality risk, independent of calcification at other locations. Unraveling the distinct pathological processes that underlie differences in risk profiles and consequences of arteriosclerosis is of crucial importance to better understand the etiological pathways of arteriosclerosis. Eventually, this may aid the development of risk profile-based interventions for cardiovascular risk reduction. In addition, considering the current emphasis by the guidelines for CAC assessment, our findings highlight the importance of expanding towards personalized assessment of arterial calcification in other vessels beyond CAC.

## Supplementary information

**Additional file 1: Table S1.** Varimax Rotated Component Matrix derived from PCA. PCA = principal component analysis; HDL = inverted high-density lipoprotein cholesterol. Bold values represent highest factor loadings per component. **Table S2.** Calcification at different locations and the risk of all-cause mortality, cardiovascular and noncardiovascular mortality among women and men. Adjusted for age, cohort, scanner, and calcification at all locations. CAC, coronary artery calcification; AAC, aortic arch calcification; ECAC, extracranial carotid artery calcification; ICAC, intracranial carotid artery calcification; VBAC, vertebrobasilar artery calcification; AVC, aortic valve calcification. Values represent hazard ratios (95%-confidence intervals) for a higher burden of each component and for the upper quartile versus lowest three quartiles (CAC, AAC, ECAC, ICAC, AVC) or the presence of calcification (VBAC). **Table S3.** Varimax Rotated Component Matrix derived from PCA excluding participants with history of cardiovascular disease. PCA = principal component analysis; HDL = inverted high-density lipoprotein cholesterol. Bold values represent highest factor loadings per component. **Table S4.** Calcification at different locations and the risk of mortality excluding participants with history of cardiovascular disease. Adjusted for age, cohort, scanner, body mass index, systolic blood pressure, diastolic blood pressure, smoking status, glucose, total cholesterol, HDL-cholesterol, and antidiabetic therapy, blood pressure, and/or lipid lowering medication use. CAC, coronary artery calcification; AAC, aortic arch calcification; ECAC, extracranial carotid artery calcification; ICAC, intracranial carotid artery calcification; VBAC, vertebrobasilar artery calcification; AVC, aortic valve calcification. Values represent hazard ratios (95%-confidence intervals) for a higher burden of each component and for the upper quartile versus lowest three quartiles (CAC, AAC, ECAC, ICAC, AVC) or the presence (VBAC) of calcification. **Figure S1A.** Risk factor profiles and calcification (upper quartile vs lowest three quartiles or presence of calcification) at different locations, among women without history of cardiovascular disease. Values represent odds ratio and 95%-confidence intervals (OR (95%-CI)) for severe versus nonsevere CAC, AAC, ECAC, ICAC, and AVC, and for the presence of VBAC. Figures are adjusted for age, cohort, scanner, antidiabetic therapy, blood pressure, and/or lipid lowering medication use, and history of cardiovascular disease. **Figure S1B.** Risk factor profiles and calcification (upper quartile vs lowest three quartiles or presence of calcification) at different locations, among men. Values represent odds ratio and 95%-confidence intervals (OR (95%-CI)) for severe versus nonsevere CAC, AAC, ECAC, ICAC, and AVC, and for the presence of VBAC. Figures are adjusted for age, cohort, scanner, antidiabetic therapy, blood pressure, and/or lipid lowering medication use, and history of cardiovascular disease.

## Data Availability

Because of data protection standards of the informed consent procedure of the Rotterdam Study, data cannot be made freely available in publicly available repositories.

## Abbreviations

AAC: Aortic arch calcification
AVC: Aortic valve calcification
BMI: Body mass index
CAC: Coronary artery calcification
ECAC: Extracranial carotid artery calcification
ICAC: Intracranial carotid artery calcification
MDCT: Multidetector computed tomography
PCA: Principal component analysis
VBAC: Vertebrobasilar artery calcification
WHR: Waist-to-hip ratio
