# Supplementary material for: Arterial calcification at multiple sites: sex-specific cardiovascular risk profiles and mortality risk—the Rotterdam Study
Source: BMC Med. 2020 Sep 24;18:263. doi: 10.1186/s12916-020-01722-7 (PMC7513304; doi:10.1186/s12916-020-01722-7)
Supplement: Supplementary file 1 — Additional file 1: Table S1. Varimax Rotated Component Matrix derived from PCA. PCA = principal component analysis; HDL = inverted high-density lipoprotein cholesterol. Bold values represent highest factor loadings per component. Table S2. Calcification at different locations and the risk of all-cause mortality, cardiovascular and noncardiovascular mortality among women and men. Adjusted for age, cohort, scanner, and calcification at all locations. CAC, coronary artery calcification; AAC, aortic arch calcification; ECAC, extracranial carotid artery calcification; ICAC, intracranial carotid artery calcification; VBAC, vertebrobasilar artery calcification; AVC, aortic valve calcification. Values represent hazard ratios (95%-confidence intervals) for a higher burden of each component and for the upper quartile versus lowest three quartiles (CAC, AAC, ECAC, ICAC, AVC) or the presence of calcification (VBAC). Table S3. Varimax Rotated Component Matrix derived from PCA excluding participants with history of cardiovascular disease. PCA = principal component analysis; HDL = inverted high-density lipoprotein cholesterol. Bold values represent highest factor loadings per component. Table S4. Calcification at different locations and the risk of mortality excluding participants with history of cardiovascular disease. Adjusted for age, cohort, scanner, body mass index, systolic blood pressure, diastolic blood pressure, smoking status, glucose, total cholesterol, HDL-cholesterol, and antidiabetic therapy, blood pressure, and/or lipid lowering medication use. CAC, coronary artery calcification; AAC, aortic arch calcification; ECAC, extracranial carotid artery calcification; ICAC, intracranial carotid artery calcification; VBAC, vertebrobasilar artery calcification; AVC, aortic valve calcification. Values represent hazard ratios (95%-confidence intervals) for a higher burden of each component and for the upper quartile versus lowest three quartiles (CAC, AAC, ECAC, ICAC, AVC) or th [file 12916_2020_1722_MOESM1_ESM.zip › Additional file 1R1.pdf]

Additional file 1

Tables: Table S1. Varimax Rotated Component Matrix derived from PCA

Table S2. Calcification at different locations and the risk of all-cause mortality, cardiovascular and noncardiovascular mortality among women and men

Table S3. Varimax Rotated Component Matrix derived from PCA excluding participants with history of cardiovascular disease

Table S4. Calcification at different locations and the risk of all-cause mortality excluding participants with history of cardiovascular disease

Figures: Fig. S1A. Risk factor profiles and severe calcification (upper quartile vs lowest three) at different atherosclerotic locations in women without history of cardiovascular disease.

Fig. S1B. Risk factor profiles and severe calcification (upper quartile vs lowest three) at different atherosclerotic locations in men without history of cardiovascular disease

Table S1. Varimax Rotated Component Matrix derived from PCA

	Women			Men		
	**Component 1**	**Component 2**	**Component 3**	**Component 1**	**Component 2**	**Component 3**
Body mass index (kg/m^2)	**0.783**	0.001	-0.091	**0.827**	-0.008	0.080
Waist-to-hip ratio	**0.717**	0.090	0.099	**0.819**	0.098	0.198
Smoking (cigarettes per day)	-0.038	-0.080	**0.778**	0.125	-0.056	**0.487**
Systolic blood pressure (mmHg)	0.107	**0.953**	-0.020	0.043	**0.947**	-0.019
Pulse pressure (mmHg)	0.084	**0.961**	0.014	-0.001	**0.952**	-0.076
Glucose (mmol/L)	**0.621**	0.110	0.022	**0.394**	0.180	**-0.382**
Total cholesterol (mmol/L)	-0.159	-0.065	**-0.627**	-0.108	0.035	**0.822**
HDL cholesterol (mmol/L)	**0.581**	0.036	0.281	**0.579**	-0.094	-0.285
Eigenvalue	2.258	1.587	1.014	2.018	1.747	1.161
Cumulative variance explained %	28.2	48.1	60.7	25.2	47.1	61.6

PCA = principal component analysis; HDL = inverted high-density lipoprotein cholesterol

Bold values represent highest factor loadings per component

Table S2. Calcification at different locations and the risk of all-cause mortality, cardiovascular and noncardiovascular mortality among women and men

	All-cause mortality	Cardiovascular mortality	Noncardiovascular mortality
**Women**	(N_{cases}/N_{atrisk}=193/1,239)	(Ncases/Natrisk= 64/1,239)	(Ncases/Natrisk=129/1,239)
CAC	1.17 (0.83-1.64)	1.00 (0.56-1.78)	1.28 (0.85-1.94)
AAC	1.33 (0.94-1.90)	1.04 (0.56-1.91)	1.52 (0.99-2.32)
ECAC	1.39 (0.99-1.96)	1.91 (1.06-3.34)	1.18 (0.77-1.81)
ICAC	1.27 (0.89-1.81)	1.83 (0.99-3.37)	1.06 (0.69-1.65)
VBAC	1.13 (0.81-1.57)	1.05 (0.59-1.85)	1.18 (0.78-1.78)
AVC	1.03 (0.75-1.41)	1.75 (1.02-3.00)	0.78 (0.52-1.15)
**Men**	(N_{cases}/N_{atrisk}=259/1,118)	(Ncases/Natrisk=70/1,118)	(Ncases/Natrisk=189/1,118)
CAC	1.16 (0.87-1.56)	2.04 (1.17-3.56)	0.93 (0.66-1.32)
AAC	1.46 (1.08-1.96)	1.45 (0.81-2.60)	1.46 (1.03-2.07)
ECAC	1.27 (0.94-1.70)	1.30 (0.74-2.27)	1.26 (0.88-1.78)
ICAC	1.06 (0.78-1.45)	1.54 (0.85-2.77)	0.92 (0.63-1.32)
VBAC	1.38 (1.04-1.82)	1.07 (0.62-1.82)	1.51 (1.09-2.10)
AVC	1.05 (0.80-1.37)	1.31 (0.80-2.16)	0.97 (0.70-1.33)

Adjusted for age, cohort, scanner, and calcification at all locations.

CAC, coronary artery calcification; AAC, aortic arch calcification; ECAC, extracranial carotid artery calcification; ICAC, intracranial carotid artery calcification; VBAC, vertebrobasilar artery calcification; AVC, aortic valve calcification.

Values represent hazard ratios (95%-confidence intervals) for a higher burden of each component and for the upper quartile versus lowest three quartiles (CAC, AAC, ECAC, ICAC, AVC) or the presence of calcification (VBAC).

Table S3. Varimax Rotated Component Matrix derived from PCA excluding participants with history of cardiovascular disease

	**Women**			**Men**		
	**Component 1**	**Component 2**	**Component 3**	**Component 1**	**Component 2**	**Component 3**
Body mass index (kg/m^2)	**0.774**	0.024	-0.078	**0.836**	0.000	0.101
Waist-to-hip ratio	**0.727**	0.103	0.068	**0.816**	0.111	0.231
Smoking (cigarettes per day)	-0.020	-0.049	**0.782**	0.090	-0.038	**0.475**
Systolic blood pressure (mmHg)	0.115	**0.954**	-0.024	0.055	**0.944**	-0.028
Pulse pressure (mmHg)	0.089	**0.960**	0.001	0.008	**0.954**	-0.052
Glucose (mmol/L)	**0.629**	0.107	0.042	**0.406**	0.179	**-0.325**
Total cholesterol (mmol/L)	-0.149	-0.028	**-0.646**	-0.100	0.009	**0.832**
HDL cholesterol (mmol/L)	**0.605**	0.019	0.226	**0.591**	-0.115	-0.294
Eigenvalue	2.288	1.580	1.013	2.048	1.728	1.161
Cumulative variance explained %	28.6	48.4	61.0	25.6	47.2	61.7

PCA = principal component analysis; HDL = inverted high-density lipoprotein cholesterol

Bold values represent highest factor loadings per component

Table S4. Calcification at different locations and the risk of mortality excluding participants with history of cardiovascular disease

	All-cause mortality	Cardiovascular mortality	Noncardiovascular mortality
	HR (95%-CI) for upper quartile versus lowest three quartiles of calcification		
**Women**	(N_{cases}/N_{atrisk}=165/1,165)	(Ncases/Natrisk= 53/1,165)	(Ncases/Natrisk=112/1,165)
CAC	1.32 (0.95-1.84)	1.47 (0.83-2.62)	1.26 (0.84-1.89)
AAC	1.39 (0.99-1.95)	1.39 (0.76-2.51)	1.39 (0.92-2.09)
ECAC	1.52 (1.09-2.12)	1.96 (1.10-3.50)	1.35 (0.89-2.03)
ICAC	1.31 (0.92-1.85)	2.19 (1.18-4.07)	1.02 (0.66-1.57)
VBAC	1.13 (0.78-1.62)	1.54 (0.84-2.82)	0.96 (0.61-1.51)
AVC	1.20 (0.87-1.67)	1.77 (1.01-3.12)	0.99 (0.66-1.49)
**Men**	(N_{cases}/N_{atrisk}=191/961)	(Ncases/Natrisk=38/961)	(Ncases/Natrisk=153/961)
CAC	1.25 (0.90-1.73)	2.52 (1.30-4.90)	1.03 (0.70-1.51)
AAC	1.35 (0.97-1.87)	2.35 (1.14-4.84)	1.16 (0.80-1.69)
ECAC	1.27 (0.92-1.74)	1.53 (0.77-3.06)	1.22 (0.85-1.75)
ICAC	1.12 (0.82-1.54)	1.39 (0.70-2.75)	1.06 (0.74-1.52)
VBAC	1.48 (1.07-2.03)	0.93 (0.43-2.01)	1.64 (1.15-2.33)
AVC	1.15 (0.83-1.58)	1.26 (0.62-2.54)	1.12 (0.78-1.61)

Adjusted for age, cohort, scanner, body mass index, systolic blood pressure, diastolic blood pressure, smoking status, glucose, total cholesterol, HDL-cholesterol, and antidiabetic therapy, blood pressure, and/or lipid lowering medication use. CAC, coronary artery calcification; AAC, aortic arch calcification; ECAC, extracranial carotid artery calcification; ICAC, intracranial carotid artery calcification; VBAC, vertebrobasilar artery calcification; AVC, aortic valve calcification.

Values represent hazard ratios (95%-confidence intervals) for a higher burden of each component and for the upper quartile versus lowest three quartiles (CAC, AAC, ECAC, ICAC, AVC) or the presence (VBAC) of calcification.

Fig. S1A. Risk factor profiles and calcification (upper quartile vs lowest three quartiles or presence of calcification) at different locations, among women without history of cardiovascular disease

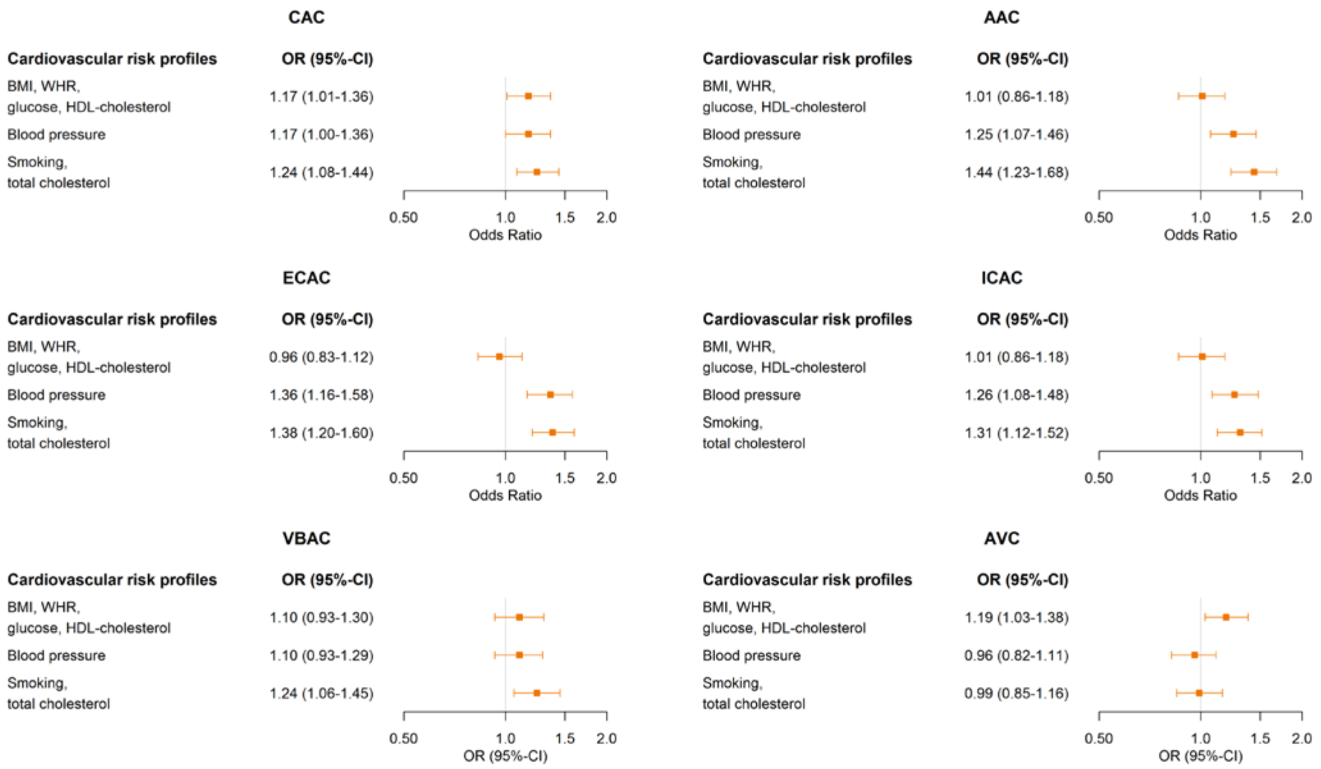

Values represent odds ratio and 95%-confidence intervals (OR (95%-CI)) for severe versus nonsevere CAC, AAC, ECAC, ICAC, and AVC, and for the presence of VBAC. Figures are adjusted for age, cohort, scanner, antidiabetic therapy, blood pressure, and/or lipid lowering medication use, and history of cardiovascular disease.

Fig. S1B. Risk factor profiles and calcification (upper quartile vs lowest three quartiles or presence of calcification) at different locations, among men

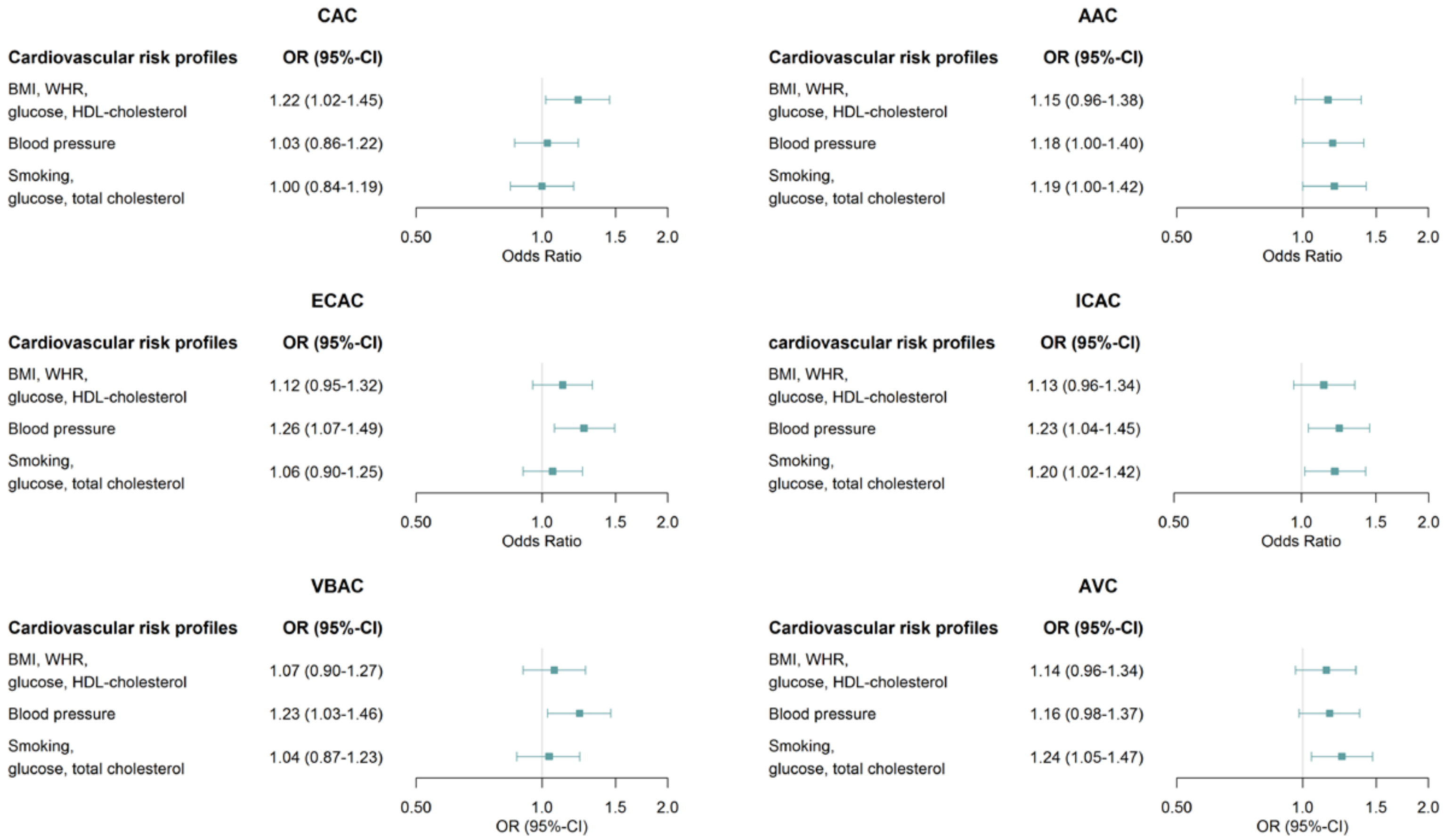

Values represent odds ratio and 95%-confidence intervals (OR (95%-CI)) for severe versus nonsevere CAC, AAC, ECAC, ICAC, and AVC, and for the presence of VBAC. Figures are adjusted for age, cohort, scanner, antidiabetic therapy, blood pressure, and/or lipid lowering medication use, and history of cardiovascular disease.
