# Supplementary figures and images for: Arterial calcification at multiple sites: sex-specific cardiovascular risk profiles and mortality risk—the Rotterdam Study
Source: BMC Med. 2020 Sep 24;18:263. doi: 10.1186/s12916-020-01722-7 (PMC7513304; doi:10.1186/s12916-020-01722-7)

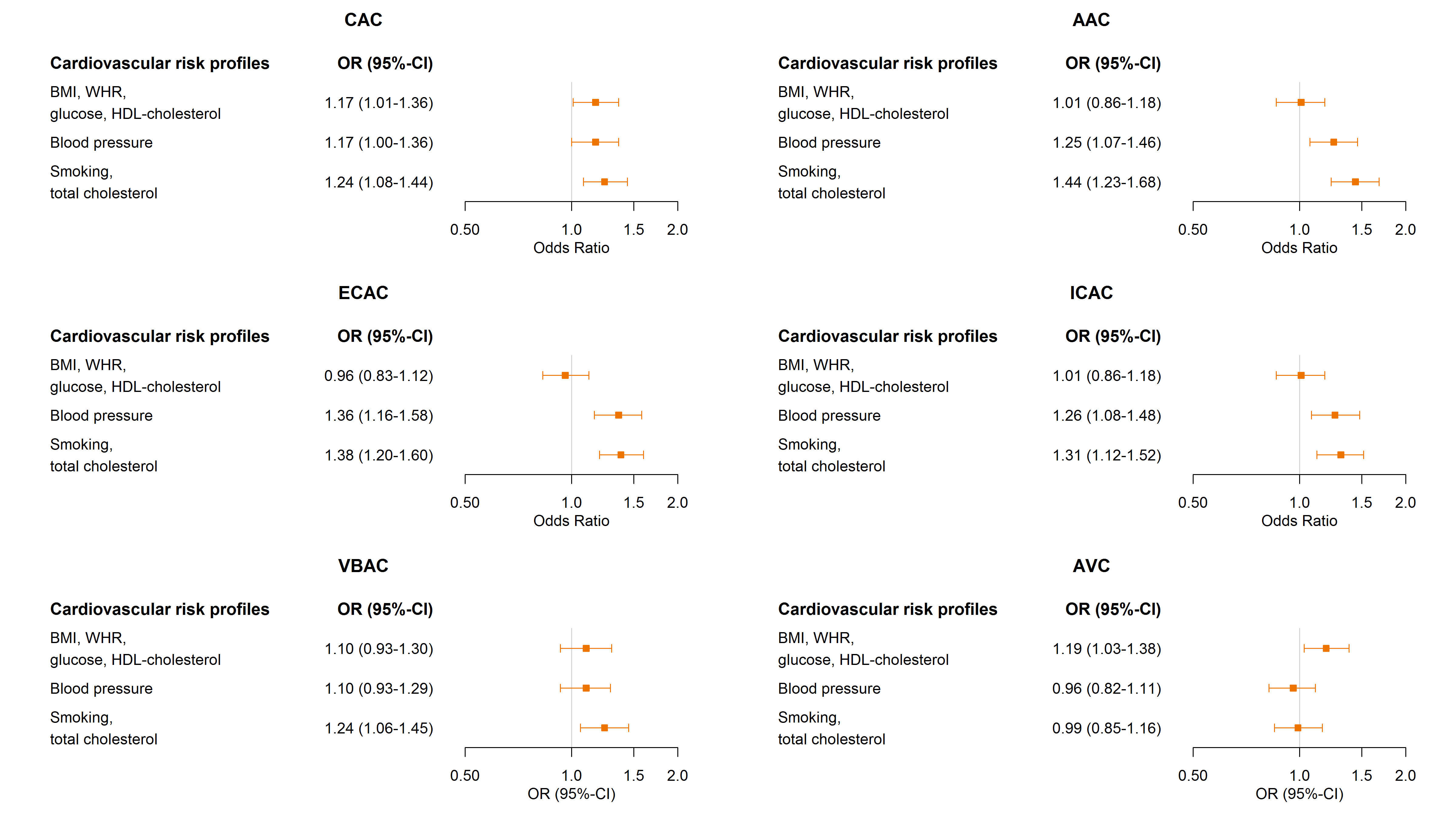

Supplement: Supplementary file 1 — Additional file 1: Table S1. Varimax Rotated Component Matrix derived from PCA. PCA = principal component analysis; HDL = inverted high-density lipoprotein cholesterol. Bold values represent highest factor loadings per component. Table S2. Calcification at different locations and the risk of all-cause mortality, cardiovascular and noncardiovascular mortality among women and men. Adjusted for age, cohort, scanner, and calcification at all locations. CAC, coronary artery calcification; AAC, aortic arch calcification; ECAC, extracranial carotid artery calcification; ICAC, intracranial carotid artery calcification; VBAC, vertebrobasilar artery calcification; AVC, aortic valve calcification. Values represent hazard ratios (95%-confidence intervals) for a higher burden of each component and for the upper quartile versus lowest three quartiles (CAC, AAC, ECAC, ICAC, AVC) or the presence of calcification (VBAC). Table S3. Varimax Rotated Component Matrix derived from PCA excluding participants with history of cardiovascular disease. PCA = principal component analysis; HDL = inverted high-density lipoprotein cholesterol. Bold values represent highest factor loadings per component. Table S4. Calcification at different locations and the risk of mortality excluding participants with history of cardiovascular disease. Adjusted for age, cohort, scanner, body mass index, systolic blood pressure, diastolic blood pressure, smoking status, glucose, total cholesterol, HDL-cholesterol, and antidiabetic therapy, blood pressure, and/or lipid lowering medication use. CAC, coronary artery calcification; AAC, aortic arch calcification; ECAC, extracranial carotid artery calcification; ICAC, intracranial carotid artery calcification; VBAC, vertebrobasilar artery calcification; AVC, aortic valve calcification. Values represent hazard ratios (95%-confidence intervals) for a higher burden of each component and for the upper quartile versus lowest three quartiles (CAC, AAC, ECAC, ICAC, AVC) or th [file 12916_2020_1722_MOESM1_ESM.zip › Figure S1AR1.tiff]

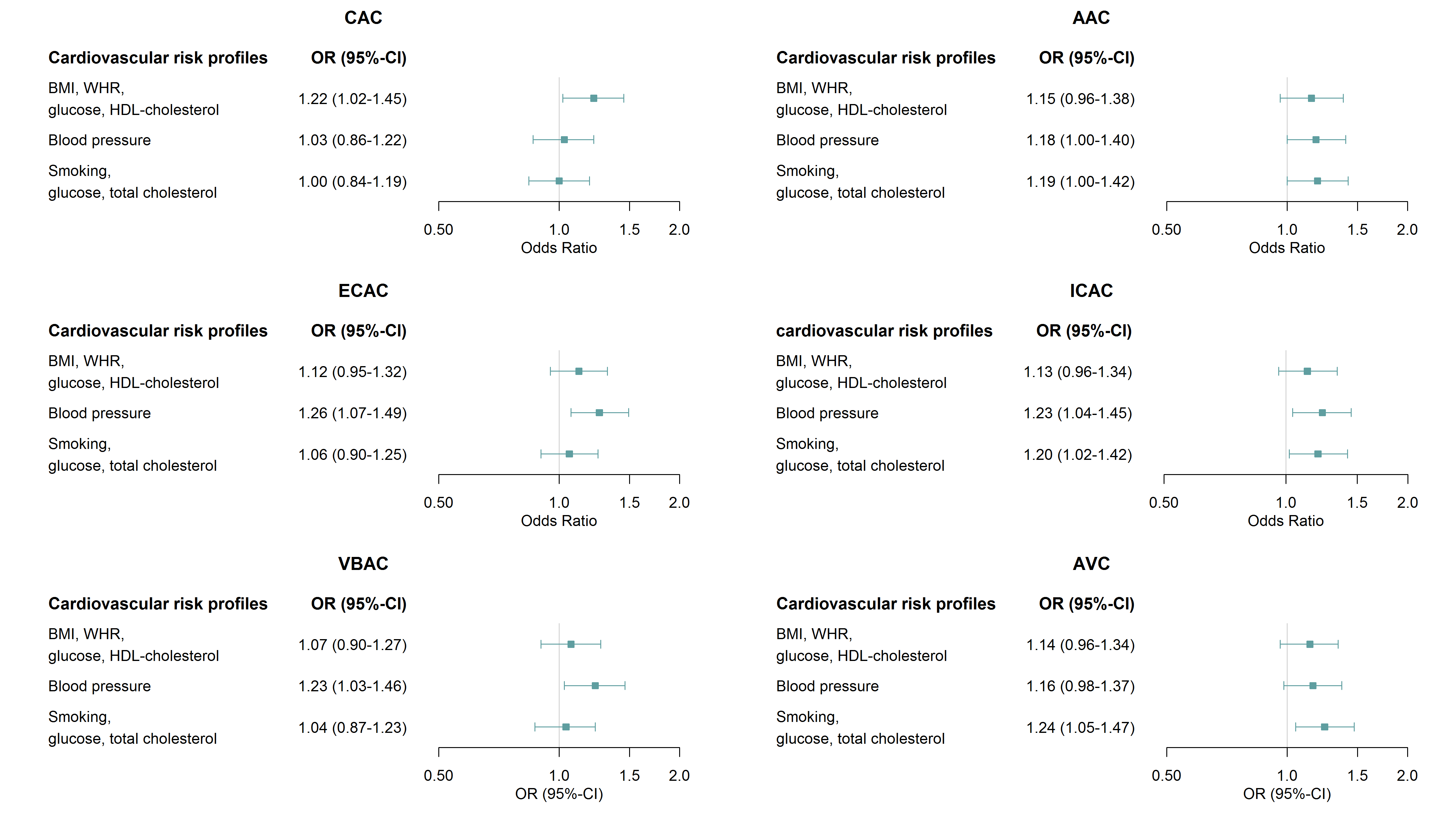

Supplement: Supplementary file 1 — Additional file 1: Table S1. Varimax Rotated Component Matrix derived from PCA. PCA = principal component analysis; HDL = inverted high-density lipoprotein cholesterol. Bold values represent highest factor loadings per component. Table S2. Calcification at different locations and the risk of all-cause mortality, cardiovascular and noncardiovascular mortality among women and men. Adjusted for age, cohort, scanner, and calcification at all locations. CAC, coronary artery calcification; AAC, aortic arch calcification; ECAC, extracranial carotid artery calcification; ICAC, intracranial carotid artery calcification; VBAC, vertebrobasilar artery calcification; AVC, aortic valve calcification. Values represent hazard ratios (95%-confidence intervals) for a higher burden of each component and for the upper quartile versus lowest three quartiles (CAC, AAC, ECAC, ICAC, AVC) or the presence of calcification (VBAC). Table S3. Varimax Rotated Component Matrix derived from PCA excluding participants with history of cardiovascular disease. PCA = principal component analysis; HDL = inverted high-density lipoprotein cholesterol. Bold values represent highest factor loadings per component. Table S4. Calcification at different locations and the risk of mortality excluding participants with history of cardiovascular disease. Adjusted for age, cohort, scanner, body mass index, systolic blood pressure, diastolic blood pressure, smoking status, glucose, total cholesterol, HDL-cholesterol, and antidiabetic therapy, blood pressure, and/or lipid lowering medication use. CAC, coronary artery calcification; AAC, aortic arch calcification; ECAC, extracranial carotid artery calcification; ICAC, intracranial carotid artery calcification; VBAC, vertebrobasilar artery calcification; AVC, aortic valve calcification. Values represent hazard ratios (95%-confidence intervals) for a higher burden of each component and for the upper quartile versus lowest three quartiles (CAC, AAC, ECAC, ICAC, AVC) or th [file 12916_2020_1722_MOESM1_ESM.zip › Figure S1BR1.tiff]
